# Temporal dynamics of *Plasmodium falciparum* population in Metehara, east-central Ethiopia

**DOI:** 10.1186/s12936-022-04277-5

**Published:** 2022-09-15

**Authors:** Abeba Gebretsadik Reda, Alebachew Messele, Hussein Mohammed, Ashenafi Assefa, Lemu Golassa, Hassen Mamo

**Affiliations:** 1 Department of Microbial, Cellular and Molecular Biology, College of Natural and Computational Sciences, Addis Ababa University, Addis Ababa, Ethiopia; 2 Aklilu Lemma Institute of Pathobiology, Addis Ababa University, Addis Ababa, Ethiopia; 3 Malaria and Neglected Tropical Diseases Research Team, Ethiopian Public Health Institute, Addis Ababa, Ethiopia

**Keywords:** *Merozoite surface proteins* 1 and 2, *Glutamate-rich protein*, Genetic diversity, Multiplicity of infection, Heterozygosity, Allelic family, Gene, Allele, *Plasmodium falciparum*

## Abstract

**Background:**

*Plasmodium falciparum* is the most serious, genetically most complex and fastest-evolving malaria parasite. Information on genetic diversity of this parasite would guide policy decision and malaria elimination endeavors. This study explored the temporal dynamics of *P. falciparum* population in two time points in Metehara, east-central Ethiopia.

**Methods:**

The participants were quantitative real-time polymerase chain reaction-confirmed patients who were recruited for uncomplicated falciparum malaria therapeutic efficacy test in 2015 and 2019. Dry blood spot samples were analysed by the nested PCR to genotype *P. falciparum merozoite surface protein* (*msp1*, *msp2)* and *glutamate-rich protein* (*glurp*) genes.

**Results:**

While *msp1*, *msp2* and *glurp* genotypes were successfully detected in 26(89.7%), 24(82.8%) and 14(48.3%) of 2015 samples (n = 29); the respective figures for 2019 (n = 41) were 31(68.3%), 39(95.1%), 25(61.0%). In 2015, the frequencies of K1, MAD20 and RO33 allelic families of *msp1*, and FC27 and IC/3D7 of *msp2* were 19(73.1%), 8(30.6%), 14(53.8%), 21(87.5%), 12(50.5%); and in 2019 it was 15(48.4%), 19(61.3%), 15(48.4%), 30(76.9%), 27(69.2%) respectively. MAD20 has shown dominance over both K1 and RO33 in 2019 compared to the proportion in 2015. Similarly, although FC27 remained dominant, there was shifting trend in the frequency of IC/3D7 from 50.5% in 2015 to 69.2% in 2019. The multiplicity of infection (MOI) and expected heterozygosity index (*He*) in 2015 and 2019 were respectively [1.43 ± 0.84] and [1.15 ± 0.91], 0.3 and 0.03 for *msp1*. However, there was no significant association between MOI and age or parasitaemia in both time points.

**Conclusion:**

The lower genetic diversity in *P. falciparum* population in the two time points and overall declining trend as demonstrated by the lower MOI and *He* may suggest better progress in malaria control in Metehara. But, the driving force and selective advantage of switching to MAD20 dominance over the other two *msp1* allelic families, and the dynamics within *msp2* alleles needs further investigation.

**Supplementary Information:**

The online version contains supplementary material available at 10.1186/s12936-022-04277-5.

## Background

Malaria remains among the leading deadliest diseases despite concerted efforts to eliminate it. Globally, 241 million malaria cases and 627,000 deaths occurred in 2020, which is more by 14 million cases and 69,000 deaths compared to what was reported in 2019 [1]. This rise was primarily attributed to the multifaceted COVID-19 disruption. In sub-Saharan Africa, the public health and socioeconomic burden of malaria is the heaviest [2]. Although 68% of the Ethiopian population still lives in malaria endemic areas, substantial progress was achieved and the health ministry set an ambitious plan of malaria elimination by 2030 [3]. A transition from high or moderate to low malaria transmission and eventual elimination and eradication requires continuous insight into the dynamics and genetic structure of the parasite population [4, 5].

*Plasmodium falciparum,* which is the gravest malaria parasite, is also the most challenging species because of its tremendous genetic plasticity [6] in terms of escaping diagnostics [7], therapeutics [8] and host immunity and thus vaccines [9]. It is genetically the most complex and most elastic *Plasmodium* species. Thus, continuous follow-up and dissection of the population of this parasite including systems biology approach has to be integrated into the routine malaria surveillance program and other research priorities to achieve elimination goals [10–12]. Multiplicity of infection (MOI) and expected heterozygosity (*He*) is considered useful surrogate marker of changes in malaria transmission intensity besides several non-genetic metrics [13].

In light of this, *P. falciparum* genetic diversity and population structure is commonly studied by genotyping block 2, block 3 and RII repeat regions of its *merozoite surface protein* (*msp1*, *msp2)* and *glutamate-rich protein (glurp)* genes, respectively [14]. Merozoite surface proteins (MSPs) encoded by *msp* genes, are both integral and peripheral membrane proteins on the surface of *Plasmodium* merozoites. MSP1 and MSP2 are the most abundant glychophosphatidylinositol-anchored proteins among the MSPs. These surface protein complexes are involved in multiple host–parasite interactions and are, therefore, of significant clinical and epidemiological relevance [15]. MSP1 which is 190-kDa is the most abundant MSP protein and has a major role in erythrocyte invasion, and is among leading blood-stage malaria vaccine candidates [16–19]. The *msp1* gene contains 17 blocks of sequence flanked by conserved regions block 2, and is grouped commonly into three allelic families namely K1, MDA20 and RO33 although a new allele family, MR, is also reported to occur worldwide [20]. The *msp2*, on the other hand, is a glycoprotein encoding gene consisting 5 blocks where the central block is the most polymorphic. The *msp2* alleles are grouped into two allelic families, FC27 and IC/3D7 [21]. The *glurp* gene has R0 (N-terminal nonrepetitive) and R2 (C-terminal repetitive) regions and encodes for a 220 kDa glutamate-rich protein (GLURP) antigen expressed throughout the lifecycle of the malaria parasites [22–24].

In contrast to the global efforts, the extent of *P. falciparum* genetic diversity is little investigated in Ethiopia. The present study was aimed at assessing the temporal changes in the genetic diversity of *P. falciparum* population circulating in Metehara using samples acquired in two time points, 2015 and 2019, in the backdrop of deployment of massive intervention strategies in the area and the country at large.

## Methods

### Study site

**Fig. 1:**
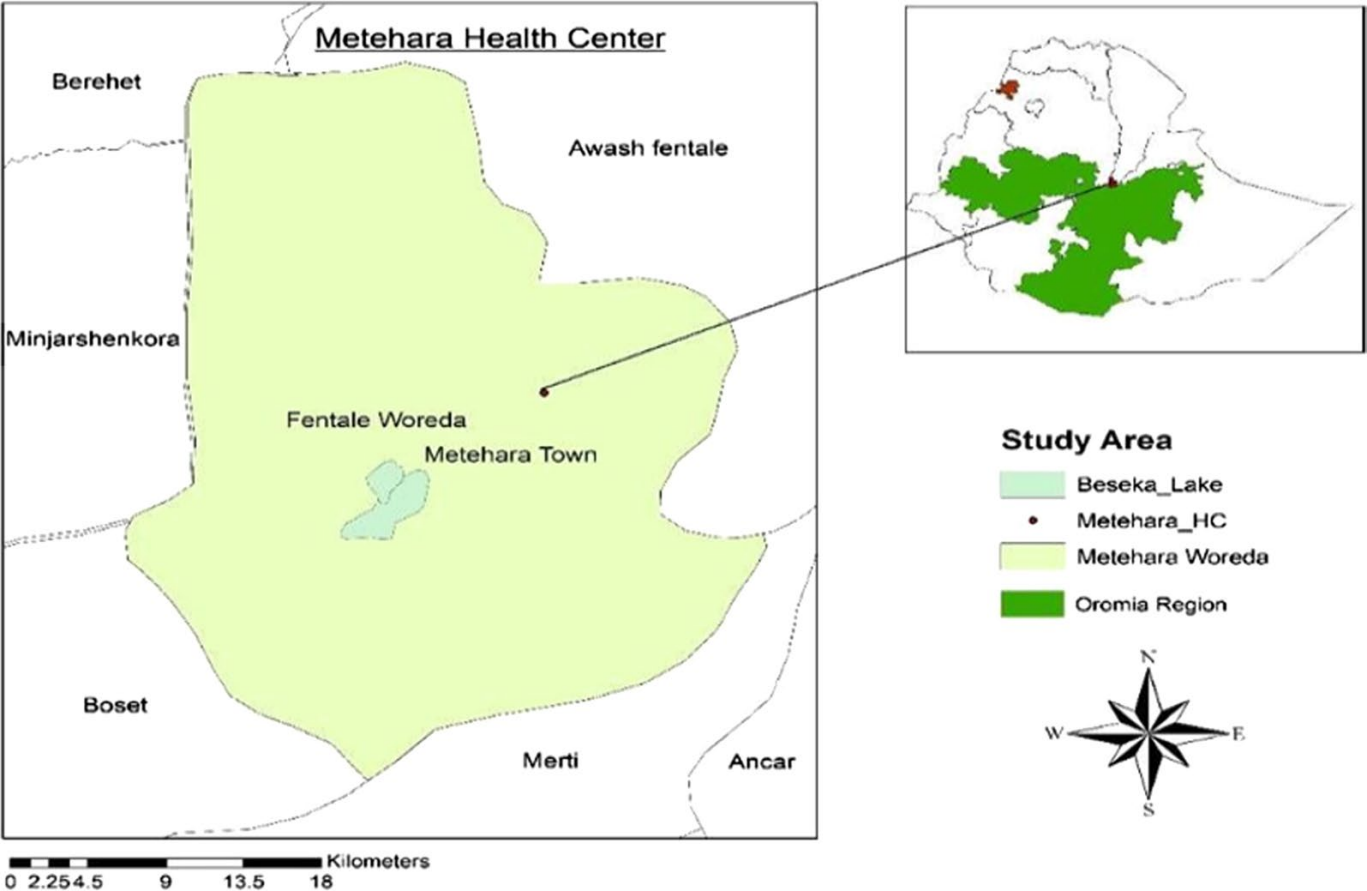
Map of Metehara the study area, showing sample collection site

Samples used for this study were collected from Metehara, a sentinel site identified for routine monitoring of the therapeutic efficacy of artemether–lumefantrine in east-central Ethiopia (Fig. 1). Metehara is located at 8°33′ N 39°16′ E situated in the rift valley area at an elevation of 947 m some 128 km to southeast of Addis Ababa. The Awash River basin provides a favorable microhabitat to support malaria vector breeding. A sugar factory estate irrigation system which depends on the nearby Awash River for the cultivation of sugarcane, and Beska River are additional breeding sites for the malaria mosquitoes.

### Study population and samples

Febrile patients having symptoms consistent with clinical malaria who were attending Metehara Health Centre were recruited; finger-prick blood samples were drawn and examined using malaria rapid detection kit (CareStart™ combo, Acces Bio, USA). Concurrently, smears were prepared and screened, and dried blood spots (DBS) were collected onto Whatman 903^®^ filter paper (Schleicher & Schuell Bio Science, Keene, NH 03431, USA) for molecular analysis. The DBS were transported, by cold chain, to Addis Ababa and stored at − 20 °C at malaria research laboratory, Ethiopian Public Health Institute (EPHI), until analysed.

### Parasite DNA extraction and infection confirmation

The molecular biology analysis of this work was done at Addis Ababa University, Aklilu Lemma Institute of Pathobiology malaria molecular laboratory. DNA was extracted from the DBS samples using the QIA amp DNA Blood Mini Kit (Qiagen Inc., Valencia, CA, USA) according to the manufacturer’s protocol. The DNA was finally eluted with a final volume of 100 µl for each sample and stored at − 20 °C until it was used for the amplification reaction. *Plasmodium falciparum* mono-infection was confirmed by the quantitative real-time polymerase chain reaction (qRT-PCR) and parasitaemia quantified [25]. Primers and positive controls were obtained from MR4 (now BEI Resources, Manassas, VA, USA). All PCR reaction mixtures were incubated in a thermal cycler (Perkin-Elmer Cetus PE 9600 iCycler thermal Cycler, Serial number 582BR006802 (Bio-Rad, Hercules, USA)). Primers used for genotyping *msp1* (block 2), *msp2* (block 3) and *glurp* (R2) are listed in Additional files 1, 2 and 3, respectively.

### Genotyping of *msp1, msp2* and *glurp*

For genotyping the target genes, the established nested PCR method was used [26]. Two-rounds of PCR reactions were carried out in a final volume of 20 μl. In primary round reaction, 4 μl of gDNA, 10 μl GoTaq Green Master Mix (Promega), 0.5 μl (0.5 μM) of each primer and 5 μl nuclease-free water were used. In secondary rounds, 2 μl of PCR amplicon, 7 μl nuclease-free water was added to the master mix preparation for secondary amplifications reaction. Cycling conditions for both PCR reactions were as follows: initial denaturation at 95 °C for 3 min, followed by 35 cycles for primary and 30 cycles for secondary reactions of denaturation at 95 °C for 1 min (94 °C for 1 min for the nested reaction) annealing at 58 °C for 2 min and extension at 72 °C for 2 min and; a final extension was carried out at 72 °C for 5 min. Positive (3D7) and DNA-free negative controls were included in each set of reactions.

The nested PCR products were resolved in 2% agarose gels (Caisson, Utah, USA), stained with ethidium bromide submerged in 0.5× TBE (Tris-borate EDTA) buffer electrophoresis at 120 V, 400 A for 45 min and visualized under UV trans-illumination and photographed (VersaDoc^®^, Bio-Rad, Hercules, USA) at 302 nm on gel documentation system. The sizes of DNA fragments were estimated by visual inspection using a 100 base pair (bp) DNA ladder marker (New England Biolabs. Inc, UK). Presence of more than one genotype was taken as a polyclonal infection, while a single allele was considered as a monoclonal infection [27]. For *msp1* and *msp2* alleles, fragment sizes were within 20 bp interval*,* and for *glurp* 50 bp interval [28].

### Data analysis

The statistical software SPSS (IBM SPSS Statistics for Windows, Version 20.0. Armonk, NY: IBM Corp.) was used to conduct all statistical analyses. For each sample, MOI was scored as the maximum number of alleles observed when all loci were taken into account and the average MOI was calculated for each sub-population. The allelic frequency and mean MOI of the *msp1*, *msp2* and *glurp* genes were calculated using proportions of allele comparisons and the Chi-squared tests. The MOI was compared using the Student t-test to assess the relationship between MOI, parasite density and patient age, and the spearman’s rank correlation coefficient was calculated. *P*-value < 0.05 was selected as a threshold for statistically significant differences. As a measure for genetic diversity, the expected heterozygosity (*He*) was calculated using the formula $He = \left( {\frac{n}{n - 1}} \right)\left( {1 - \sum p^{2} } \right)$ where ‘n’ is the number of isolates analysed and ‘p’ the frequency of each different allele at a locus [29].

## Results

### Demographic, parasitological and clinical data

Of the total participants in 2015 (n = 33), males were 17(51.5%) with a male-to-female ratio of 1.1. Males in 2019 (n = 50) were 26(52.0%) and the male-to-female ratio was also 1.1. While the mean (range) age of the participants in 2019 was 24 (3–60) year, in 2015 it was 25 (4–60). From these 83 total samples screened for *P. falciparum* mono-infection, microscopy and RDT, respectively, could detect only 58(69.9%) and 47(56.6%) of the cases. The qPCR confirmed these and identified additional missed cases by microcopy/RDT raising the number of *P. falciparum*-positives to 70(84.3%), 29 for 2015 samples and 41 for 2019. Therefore, the number of samples genotyped was 70. Asexual mean parasitaemia was 1593.00 (95% CI 992.10–2194.06) in 2015 and 3893.60 (95% CI 2286.05–5501.01) in 2019 (Table 1). The percentage of gametocytaemic patients was 3.01 and 2.06 in 2015 and 2019, respectively.

**Table 1:** Demographic, parasitological and clinical characteristics of study participants in 2015 and 2019 in Metehara, east-central Ethiopia

Characteristics	2015 (N = 33)	2019 (N = 50)
Male/female ratio	1.1	1.1
Age (year), mean ± SD	25 ± 1.7	24 ± 1.7
Age group		
< 5, no (%)	2(6.9)	6(12.0)
5–15, no (%)	7(21.2)	9(18.0)
> 15, no (%)	24(82.8)	35(70.0)
Feverish patients on day 0 (%) (axillary temperature > 37.5 °C)	27(81.8)	40(80.0)
Temperature (°C), mean (range)	38.03(36.10–41.40)	37.7(36.40–40.20)
Mean parasitaemia (parasites/µl)	1593.00 (95% CI 992.10–2194.06)	3893.60 (95% CI 2286.05–5501.01)
Gametocytaemic patients (%)	3.01	2.06

SD: standard deviation, no: number, µl: micro liter, CI: confidence interval

### Frequencies of *msp1, msp2* and *glurp* allelic families

While *msp1*, *msp2* and *glurp* genotypes were successfully detected in 26(89.7%), 24(82.8%) and 14(48.3%) of 2015 samples (n = 29), respectively; the respective figures for 2019 (n = 41) were 31(75.6%), 39(95.1%), 25(61.0%). In 2015, the frequencies of K1, MAD20 and RO33 allelic families of *msp1* were 19(73.1%), 8(30.6%) and 14(53.8%), respectively. In 2019, the frequencies of the respective alleles were 15(48.4%), 19(61.3%) and 15(48.4%). In 2015, the frequencies of FC27 and IC/3D7 allelic variants of *msp*2 were 87.5% and 50.5%, respectively. In 2019, the frequencies of the respective alleles were 76.9% and 69.2%, respectively (Table 2).

**Table 2:** Frequency of monoclonal and polyclonal infections of alleles of *msp1* and *msp2* genes of *Plasmodium falciparum* in 2015 and 2019 in Metehara, east-central Ethiopia

Gene	*msp1*							*msp2*		
Year	Allele									
	K1	MAD20	RO33	K1 + MAD20	K1 + RO33	MAD2 + RO33	K1 + MAD20 + RO33	IC/3D7	FC27	IC/3D7 + FC27
2015	10	0	4	2	4	3	3	3	12	9
2019	7	7	2	2	3	7	3	9	12	18
Total	17	7	6	4	7	10	6	12	24	27

As far as *msp1* allelic variants are concerned, among the samples analysed the frequency of K1 allele dropped from 73.1% in 2015 to 48.4% in 2019 while the frequency of MAD20 allele increased from 30.6% in 2015 to 61.3% in 2019. However, the frequency of RO33 allelic variant was 53.8% in 2015 and 50.5% in 2019. For *msp*2 allelic variants, the frequencies of FC27 and IC/3D7 alleles were 87.5% and 50.5% in 2015, respectively, and the corresponding figures in 2019 were 76.9% and 69.2%, respectively. In other words, the frequency of FC27 allele decreased from 87.5% in 2015 to 76.9% in 2019 while the frequency of IC/3D7 allele increased from 50.5% in 2015 to 69.2% in 2019. Higher proportion of *glurp* was detected in 2019 compared to the baseline. However, for all the three genes the increase or decline of the allelic families or variants was not statistically significant.

### MOI and *He*

The proportion of polyclonal infections for *msp1* were 46.6% (12/26) in 2015 and 51.6% (16/31) in 2019, and for *msp2* it was 37.5(9/24) and 46.2(18/39) respectively. Detailed monoclonal and polyclonal infections by the different allelic families in the two time points for the two genes are indicated in Tables 2, 3 and 4. The mean MOI in 2015 and 2019 were, respectively, [1.43 ± 0.84] and [1.15 ± 0.91] for *msp1*, [1.18 ± 0.67] and [1.39 ± 0.59] for *msp2*, and [0.20 ± 0.42] and [0.61 ± 0.31] for *glurp*. Similarly, *He* of *msp1*, *msp2* and *glurp* in 2015 and 2019 were respectively 0.3, 0.03; 0.56, 0.57; and 0.55, 0.61 all with statistically significant variation. Neither parasite density (Spearman rank correlation 0.162, *p* = 0.560) nor participant age (Spearman rank correlation 0.118, *p* = 0.670) was significantly associated with either *msp1* or *msp2* MOI in both 2015 and 2019 samples.

**Table 3:** Total percentage polyclonal infections for *msp1*, *msp2* genes of *Plasmodium falciparum* in 2015 and 2019 in Metehara, east-central Ethiopia

Gene	Polyclonal infection, no (%)	
	2015	2019
*msp1*	12(38.7)	15(48.4)
*msp2*	9(37.5)	18(46.2)

**Table 4:** Multiplicity of infection and expected heterozygosity index for *Plasmodium falciparum msp1*, *msp2* and *glurp* genes in Metehara, east-central Ethiopia

Year	MOI (mean ± SD)			*He*		
	*msp1*	*msp2*	*glurp*	*msp1*	*msp2*	*glurp*
2015	1.43 ± 0.84	1.18 ± 0.67	0.20 ± 0.42	0.30	0.56	0.55
2019	1.15 ± 0.91	1.39 ± 0.59	0.61 ± 0.31	0.03	0.57	0.61
Overall	1.67	–	–	0.48	0.70	0.58

*He*: heterozygosity index, MOI: multiplicity of infection, SD: standard deviation

### Allele fragment size

Fragments sizes, in bp, for *msp1* allelic families ranged for K1 (300–400), MAD20 (300–450) and RO33 (300–350); and that of *msp2* allelic families FC27 and IC/3D7 was (400–800). Eight R2 repeat region fragments of *glurp*, designated I–VIII, with size range of 501–900 bp were identified (Table 5). Genotype II (551–600) was the most frequent variant seen in both 2015 (3(30.3%)) and 2019 (4(36.3%)).

**Table 5:** Allelic size variants of *msp1* and *msp2* allelic families, and *glurp* R2 repeat region of *Plasmodium falciparum* population in 2015 and 2019 in Metehara, east-central Ethiopia

Gene and allelic family	Variant fragment size (bp)	Frequency (%)	
		2015	2019
*msp1*		26(100)	31(100)
K1	300–400	19(73.1)	15(48.4)
MAD20	300–450	8(30.8)	18(58.1)
RO33	300–350	14(53.8)	15(48.4)
*msp2*		24(100)	39(100)
ID/3D7	400–800	12(50.0)	27(69.2)
FC27	400–800	21(87.5)	30(76.9)
*glurp*		14(100)	25(100)
I	501–550	1(10.0)	1(9.1)
II	551–600	3(30.3)	4(36.3)
III	601–650	1(10.1)	1(9.1)
IV	651–700	1(10.1)	1(9.1)
V	701–750	1(10.1)	1(9.1)
VI	751–800	1(10.1)	1(9.1)
VII	801–850	1(10.1)	1(9.1)
VIII	851–900	1(10.1)	1(9.1)

bp: base pair

## Discussion

This study is the first of its kind to assess *P. falciparum* genetic diversity in two time points in a low malaria transmission area in Ethiopia. The genetic diversity of *P. falciparum* was relatively lower genetic diversity in 2019 than 2015 at least with respect to *msp1* gene. Moreover, in terms of size polymorphism, the *msp1* gene was higher in frequency than *msp2* and *glurp*. The core findings of this study regarding the relative distributions of the allelic families of the three genes, MOI, *He*, and relationship between MOI and parasitaemia or patient age are similar to some studies and depart from others that have been done in various settings of Ethiopia, sub-Saharan Africa as well as globally.

*Plasmodium falciparum* genetic diversity may vary with malaria transmission intensity and ongoing control interventions, sample size, season of sample collection, and study design including participant clinical status, and socio-demographic characteristics. Some studies focused on febrile but uncomplicated patients other involved febrile and complicated cases, yet others worked on asymptomatic participants. There are several additional field and laboratory factors that limit comparison and contrast between various studies. Therefore, *P. falciparum* genetic diversity, MOI and overall population structure data interpretation and comparisons need caution.

Previous studies from divergent settings in Ethiopia like in the southwest [30] and northwest [31–33] using variable sample size (88–118) collected during peak malaria transmission season, recorded high level (59–78%)* P. falciparum* polyclonal infections with respect to the target genes of this study. It was only in a semi-arid area in the northeast of Ethiopia that *P. falciparum* polyclonal infection was reported below 50% [34]. The overall mean MOI in these studies that reported high polyclonal infections and based on uncomplicated malaria patients ranged 1.2–2.8 and *He* 0.20–0.82. None of these same four studies documented significant relationship between MOI and patient age or parasitaemia. While allelic family K1 of *msp1* was dominant in the southwestern and two northwestern sites, MAD20 was higher from one northwestern setting. Concerning *msp2* allelic families, in three of the above sites the frequency of IC/3D7 was higher although marginally in the northwest. In the rest two sites FC27 was predominant.

In another study in the southwest Ethiopia [35], K1 was the predominant *msp1* allelic family followed by MAD20 and RO33, and *msp2* allelic family FC27 had higher frequency compared to IC/3D7 with 80% polyclonal infection and overall mean MOI of 3.2. The study reported *He* of 0.43 for *msp1* and 0.85 for *msp2* demonstrating high levels of genetic diversity and mixed-strain infections. However, it did not observe significant association between MOI and age or parasite density.

A study in east-central Ethiopia detected more frequent MAD20 allelic family of *msp1* followed by K1 and RO33 [36] and overall MOI 1.67 and *He* 0.48 indicating slightly low *P. falciparum* genetic diversity. Similarly, the distribution of IC/3D7 and FC27 allelic families of *msp2* was nearly comparable with polyclonal infection 40.5%, MOI of 1.4 and *He* 0.49 in the east-central part of the country [37]. This is considered intermediate *P. falciparum* genetic diversity. The current study, which is along the same rift valley with this previous studies, has similar MAD20 dominance suggesting possible *P. falciparum* strain connectivity. Similarly lower *P. falciparum* genetic diversity was noticed in the western edge of Ethiopia where MAD20 of *msp1* and FC27 of *msp2* reported to predominate [38].

Dominance of the *msp1* allelic family MAD20 among uncomplicated malaria cases was recorded in neighboring Sudan although the distributions of FC27 and IC1/3D7 *msp2* allelic families were approximately the same across disease severity [39]. The authors observed no statistically significant differences in MOI between different age groups although the majority of *P. falciparum* isolates from uncomplicated and severe malaria patients consisted of multiple genotypes.

In other sub-Saharan Africa countries like Congo Brazzaville [40], K1 allelic family tended to predominate followed by MAD20 and the distribution of *msp2* allelic families 3D7 and FC27 was nearly equal. The study revealed overall MOI of 2.64 with 86% polyclonal infections and no correlation between MOI age or parasite density. Moreover, a study [41] in selected sub-Saharan Africa countries with varying levels of endemicity, namely Malawi, Tanzania, Uganda, Burkina Faso and São Tomé found highly polymorphic on *P. falciparum msp1*, *msp2* and *glurp* markers with low allele frequencies but very high *He* values (0.68–0.99).

In South Africa [42], RO33 allele of *msp1* (84.8%), 3D7 allele of *msp2* (90.8%) were dominant in 2006–2007, but in the same setting in 2013–2016 polyclonal infection rates of *msp1* ranged from 76.7 to 29.1% and *msp2* from 62.4 to 28.3% with significant decline of allelic types in 2013–2016. Similarly, the MOIs for both *msp1* and *msp2* were higher in isolates in 2006–2007 than those in 2013–2016 showing dramatic reduction in *P. falciparum* genetic diversity. On the other hand, K1 of* msp1* and FC27 of *msp2* were the principal allelic families detected in Madagascar [43] although the proportions of both allelic families of both genes varied significantly between sites although genetic diversity was similar between sites and that parasite flow within sites was limited.

In West Africa African countries such as Burkina Faso, *msp1* allelic family K1 was predominant followed by MAD20 and R033 [44]. In *msp2*, the 3D7 allelic family was the most frequently detected with 93.1% compared to FC27 with 41.3%. High genetic diversity and allelic frequencies of both *msp1* and *msp2* were seen with overall mean MOI of 1.95. A latter study in Burkina Faso that genotyped *P. falciparum msp1* and *msp2* demonstrated a negative correlation between MOI and host age and parasite density, suggesting within-host competition among co-infecting genetically distinct *P. falciparum* variants [45]. Moreover, the authors reported the presence of each allelic family of the *msp1* and *msp2* genes year round with no significant monthly fluctuation.

In Cameroon [46], *msp1* K1 allelic family was the most abundant and the proportion of polyclonal infection was 60%. In Southern district of Brazzaville [47], K1 allelic family was predominant and 3D7 family was the most prevalent in the *msp2* gene. Overall, the mean MOI was 2.2 and 83% of the cases were polyclonal infections. No statistically significant correlation was seen between MOI and patient age but there was with parasite density. *Plasmodium falciparum* clinical isolates had high diversity and mainly of multiple clones. The basis for the positive association between parasite densities and MOI was discussed by the authors.

From Equatorial Guinea [48], MAD20 allelic family was very predominant (96.07%) followed by K1; and concerning *msp2*, the FC27 allelic family was the most frequently detected with (97.69%). A study [49] that examined the genetic diversity of *P. falciparum* within a household in north‑central Nigeria, reported remarkable degree of genetic diversity and polyclonal infections. Further study that analysed *P. falciparum* isolates from Nigeria and Senegal found K1 and IC/3D7 to be the most frequent *msp1* and *msp2* allelic families in both countries [50]. MOI was over 1 showing the widespread presence of polyclonal infections in both countries although more commonly in Senegal than in Nigeria.

An earlier study in Senegal [51] documented very large *P. falciparum* genetic diversity. In Senegal [52], for *msp1* gene, K1 allelic family was predominant and for *msp2* gene, IC/3D7 allelic family was the most represented. Polyclonal isolates found were 36% and 31% for *msp1* and *msp2* respectively having an overall mean MOI 2.56 showing low to intermediate genetic diversity (*He* = 0.394–0.637). A recent study that employed multiplexed amplicon deep sequencing in two sites in Senegal [53] found K1 and IC/3D7 allelic families to be the most predominant allelic families of *msp1* and *msp2* genes in both sites. The mean MOI for both genes was 3.07 and 1.76 revealing a high genetic diversity.

The genetic diversity and relative distribution of allelic frequencies of *P. falciparum msp1* and *msp2* or *glurp* genes in Southeast Asia is not much different from findings in Sub-Saharan Africa. For instance, from northern India, extensive diversity was found in *msp1* alleles with predominantly RO33 alleles and K1 the least [54]. From Malaysia, the *msp1* and *msp2* allelic families that were predominant were K1 and FC27, respectively [55]. The *glurp* genotype VI of the R2 was predominant. In the same study; MAD20, 3D7 and *glurp* genotype IV allelic families of the three genes were predominant in a different setting. A study from Indonesia [56] revealed the higher proportion of MAD20 followed by K1 and RO33 for *msp1* and 3D7 for *msp2.*

In a study conducted at China–Myanmar border region from 2006 to 2011 [57], the *msp1* gene MAD20 family was dominant, followed by the K1 and RO33 families. For the *msp2* gene, the most frequent allele was the FC27 family. A significant positive correlation between the MOI and parasite density was found in the *msp1* gene. High rate of multiple genotypes (96.5%) with overall MOI of 3.21 was observed in *P. falciparum* isolates from along the Thai-Myanmar borders [58]. The findings were different from site to site and K1 and MAD20 were predominant. The study revealed that 3D7 was the most dominant *msp2* allelic family and *glurp* gene was the least diverse. In Laos, the frequency of K1 was the highest followed by MAD20 and RO33 with high level of polyclonal infections although the MOI did not exceed 2.0 [59]. The authors did not get parasite density or age statistically significantly related to MOI. In Vietnam, one study [60] demonstrated that for *msp1*, MAD20 was the most prevalent followed by K1 allelic family, with no sample testing positive for RO33. For *msp2*, 3D7 allelic family was predominant. The MOI of *msp1* and *msp2* was 2.6 and 1.1, respectively, and the overall mean MOI was 3.7 according to the study.

Pertaining South American countries, a study from Brazil [61] that compared the temporal dynamics of *glurp* gene of *P. falciparum* in 1993 and 2008 found no differences between the two time points with regard to the frequencies of the fragment variants of the R0 and R2 regions of the gene. The authors found that polyclonal infections were less common and there was overall limited polymorphic variation of *glurp*. Similarly, the genetic diversity and allele frequencies of *msp1*, *msp2* and *glurp* genes were evaluated and detected few polyclonal infections and low genetic diversity among locally transmitted *P. falciparum* isolates in Panamá [62].

In summary, the decrease in the frequency of K1 allelic variants from 73.1% in 2015 to 48.4% in 2019 in the current study may indicate the susceptibility of the variants carrying this allele to the ongoing intervention strategies in the country as opposed to MAD20 allelic variant that increased in frequency from 30.6% in 2015 to 61.3% in 2019, suggesting possible positive selection pressure favoring this allele. On the other hand, RO33 allelic variants of *msp1* were slightly decreased from 53.8% in 2015 to 48.4% in 2019 showing probable modest susceptibility to the ongoing malaria intervention strategies. By the same token, *msp2* allelic variant FC27 decreased in frequency from 87.5% in 2015 to 76.9% in 2019 while the frequency of IC/3D7 increased from 50.5% in 2015 to 69.2% in 2019. This may suggest that malaria intervention strategies in the study area have impacted on the frequency of parasite strains carrying this allelic variant as opposed to IC/3D7 allelic variant that seems to be positively selected to populate in the study area despite the ongoing intervention strategies.

## Conclusions

This study found limited genetic diversity of *P. falciparum* isolates from the study area with most infections being monoclonal. This may be explained by the low prevalence of infection in the setting. However, there is a need for further studies in the current and similar as well as different transmission settings in the country using larger sample size in multiple seasons, and employing other more robust population genetic markers such as microsatellites and amplicon deep sequencing data in addition to  allele fragment length polymorphisms. The dynamics in the relative allelic family distribution of the MAD20 of *msp1* and IC/3D7 of *msp2* requires a longitudinally assessment.

## Supplementary Information

**Additional file 1.** Primers used for *msp1* genotyping.

**Additional file 2.** Primers used for *msp2* genotyping.

**Additional file 3.** Primers used for *glurp* genotyping.

## Data Availability

The dataset supporting the conclusion of this article is included within the article and the primers used to genotype the three genes are presented in attached Additional files 1, 2 and 3.

## Abbreviations

*He*: Expected heterozygosity
MOI: Multiplicity of infection
MSP1: Merozoite surface protein 1
MSP2: Merozoite surface protein 2
GLURP: *Glutamate-rich protein*
*msp1*: *Merozoite surface protein *1 gene
*msp2*: *Merozoite surface protein* 2 gene
*glurp*: *Glutamate-rich protein* gene
qRT-PCR: Quantitative real-time polymerase chain reaction
TBE: Tris-borate EDTA

## References

[1] WHO (2021). World malaria report.

[2] WHO (2017). World malaria report.

[3] Federal Democratic Republic of Ethiopia Ministry of Health (2020). National strategic plan for malaria prevention, control and elimination in Ethiopia, 2021–2025.

[4] Cotter C, Sturrock HJ, Hsiang MS, Liu J, Phillips AA, Hwang J (2013). The changing epidemiology of malaria elimination: new strategies for new challenges. Lancet.

[5] WHO (2018). Disease surveillance for malaria elimination: an operational manual.

[6] Walliker D, Quakyi IA, Wellems TE, Mccutcha TF, Szarfman A, London WT (1987). Genetic analysis of the human malaria parasite *Plasmodium falciparum*. Science.

[7] Feleke SM, Reichert EN, Aydemir O, Mohammed H, Brhane BG, Mamo H (2021). *Plasmodium falciparum* is evolving to escape malaria rapid diagnostic tests in Ethiopia. Nat Microbiol.

[8] Haldar K, Bhattacharjee S, Safeukui I (2018). Drug resistance in *Plasmodium*. Nat Rev Microbiol.

[9] Pance A (2020). Diversify and conquer: the vaccine escapism of *Plasmodium falciparum*. Microorganisms.

[10] Roch LKG, Chung DD-W, Ponts N (2012). Genomics and integrated systems biology in *Plasmodium falciparum*: a path to malaria control and eradication. Parasite Immunol.

[11] Viriyakosol S, Siripoon N, Petcharapirat C, Petcharapirat P, Jarra W, Thaithong S (1995). Genotyping of *Plasmodium falciparum* isolates by the polymerase chain reaction and potential uses in epidemiological studies. Bull World Health Org.

[12] Dalmat R, Naughton B, Kwan-Gett TS, Slyker J, Stuckey EM (2019). Use cases for genetic epidemiology in malaria elimination. Malar J.

[13] Tusting LS, Bousema T, Smith DL, Drakeley C (2014). Measuring changes in *Plasmodium falciparum* transmission: precision, accuracy and costs of metrics. Adv Parasitol.

[14] Medicines for Malaria Venture & World Health Organization. Methods and techniques for clinical trials on antimalarial drug efficacy: genotyping to identify parasite populations. Informal consultation organized by MMV and WHO, 29–31 May 2007, Amsterdam, The Netherlands.

[15] Beeson JG, Drew DR, Boyle MJ, Feng G, Fowkes FJ, Richards JS (2016). Merozoite surface proteins in red blood cell invasion, immunity and vaccines against malaria. FEMS Microbiol Rev.

[16] Holder AA, Blackman MJ, Burghaus PA, Chappel JA, Ling IT, Mcallum-Deighton N, Shai S (1992). A malaria merozoite surface protein (MSP-1)—structure, processing and function. Mem Inst Oswaldo Cruz.

[17] Miller LH, Roberts T, Shahabuddin M, McCutchan TF (1993). Analysis of sequence diversity in the *Plasmodium falciparum* merozoite surface protein-1 (MSP-1). Mol Biochem Parasitol.

[18] Chitarra V, Holm I, Graham A (1999). The crystal structure of C-terminal merozoite surface protein 1 at 1.8 resolution, a high protective malaria vaccine candidate. Mol Cell.

[19] Dijkman PM, Marzluf T, Zhang Y, Chang S-YS, Helm D, Lanzer M (2021). Structure of the merozoite surface protein 1 from *Plasmodium falciparum*. Sci Adv.

[20] Takala SL, Escalante AA, Brancha OH, Kariukid S, Biswase S, Chaiyarojf SC (2006). Genetic diversity in the Block 2 region of the merozoite surface protein 1 (MSP-1) of *Plasmodium falciparum*: additional complexity and selection and convergence in fragment size polymorphism. Infect Genet Evol.

[21] Ferreira MU, Hartl DL (2007). *Plasmodium falciparum*: worldwide sequence diversity and evolution of the malaria vaccine candidate merozoite surface protein-2 (MSP-2). Exp Parasitol.

[22] Borre MB, Dziegiel M, Høgh B, Petersen E, Rieneck K, Riley E (1991). Primary structure and localization of a conserved immunogenic *Plasmodium falciparum* glutamate rich protein (GLURP) expressed in both the preerythrocytic and erythrocytic stages of the vertebrate life cycle. Mol Biochem Parasitol.

[23] Høgh B, Thompson R, Zakiuddin IS, Boudin C, Borre M (1993). Glutamate rich *Plasmodium falciparum* antigen (GLURP). Parassitologia.

[24] de Stricker K, Vuust J, Jepsen S, Oeuvray C, Theisen M (2000). Conservation and heterogeneity of the glutamate-rich protein (GLURP) among field isolates and laboratory lines of *Plasmodium falciparum*. Mol Biochem Parasitol.

[25] Rougemont M, Van Saanen M, Sahli R, Hinrikson HP, Bille J, Jaton K (2004). Detection of four *Plasmodium* species in blood from humans by 18S rRNA gene subunit-based and species-specific real-time PCR assays. J Clin Microbiol.

[26] Snounou G (2002). Genotyping of *Plasmodium* spp. nested PCR. Methods Mol Med.

[27] Zhong D, Koepfli C, Cui L, Yan G (2018). Molecular approaches to determine the multiplicity of *Plasmodium* infections. Malar J.

[28] Tanabe K, Mackay M, Goman M, Scaife JG (1987). Allelic dimorphism in a surface antigen gene of the malaria parasite *Plasmodium falciparum*. J Mol Biol.

[29] Nei M (1978). Estimation of average heterozygosity and genetic distance from a small number of individuals. Genetics.

[30] Mohammed H, Mindaye T, Belayneh M, Kassa M, Assefa A, Tadesse M (2015). Genetic diversity of *Plasmodium falciparum* isolates based on msp-1 and msp-2 genes from Kolla-Shele area, Arbaminch Zuria District, South West Ethiopia. Malar J.

[31] Mohammed H, Kassa M, Assefa A, Tadesse M, Kebede A (2017). Genetic polymorphism of merozoite surface protein-2 (MSP-2) in *Plasmodium falciparum* isolates from Pawe, northwest Ethiopia. PLoS ONE.

[32] Mohammed H, Kassa M, Mekete K, Assefa A, Taye G, Commons RJ (2018). Genetic diversity of the msp-1, msp-2, and glurp genes of *Plasmodium falciparum* isolates in northwest Ethiopia. Malar J.

[33] Mohammed M, Hassen K, Assefa A, Mekete K, Tadesse G, Taye G (2019). Genetic diversity of *Plasmodium falciparum* isolates from patients with uncomplicated and severe malaria based on msp-1 and msp-2 genes in Gublak, northwest Ethiopia. Malar J.

[34] Mohammed H, Assefa A, Chernet M, Wuletaw Y, Commons RJ (2021). Genetic polymorphisms of *Plasmodium falciparum* isolates from Melka-Werer, North East Ethiopia based on the merozoite surface protein-2 (msp-2) gene as a molecular marker. Malar J.

[35] Abamecha A, El-Abid H, Yilma D, Addisu W, Ibenthal A, Bayih AG (2020). Genetic diversity and genotype multiplicity of *Plasmodium falciparum* infection in patients with uncomplicated malaria in Chewaka district, Ethiopia. Malar J.

[36] File T, Chekol T, Solomon G, Dinka H, Golassa L (2021). Detection of high frequency of MAD20 allelic variants of *Plasmodium falciparum* merozoite surface protein 1 gene from Adama and its surroundings, Oromia, Ethiopia. Malar J.

[37] File T, Golassa L, Dinka H (2022). *Plasmodium falciparum* clinical isolates reveal analogous circulation of 3D7 and FC27 allelic variants and multiplicity of infection in urban and rural settings: the case of Adama and its surroundings, Oromia, Ethiopia. J Parasitol Res.

[38] Tadele G, Jatieh FK, Oboh M, Oriero E, Dugassa S, Amambua-Ngwa A, et al. Low genetic diversity of *Plasmodium falciparum* merozoite surface protein 1 and 2 and multiplicity of infections in western Ethiopia following effective malaria interventions. Preprint 10.21203/rs.3.rs-1385055/v1. 10.1186/s12936-022-04394-1 PMC9753253 36522733

[39] Abdel Hamid MM, Elamin AF, Albsheer MMA, Abdalla AAA, Mahgoub NS, Mustafa SO (2016). Multiplicity of infection and genetic diversity of *Plasmodium falciparum* isolates from patients with uncomplicated and severe malaria in Gezira State, Sudan. Parasit Vectors.

[40] Singana BP, Mayengue PI, Niama RF, Ndounga M (2019). Genetic diversity of *Plasmodium falciparum* infection among children with uncomplicated malaria living in Pointe-Noire, Republic of Congo. Pan Afr Med J.

[41] Mwingira F, Nkwengulila G, Schoepflin S, Sumari D, Beck H-P, Snounou G (2011). *Plasmodium falciparum* msp1, msp2 and glurp allele frequency and diversity in sub-Saharan Africa. Malar J.

[42] Huang BO, Tuo F, Liang Y, Wu W, Wu G, Huang S (2018). Temporal changes in genetic diversity of msp1, msp2, and msp3 in *Plasmodium falciparum* isolates from Grande Comore Island after introduction of ACT. Malar J.

[43] Ralinoro F, Rakotomanga TA, Rakotosaona R, Rakoto DAD, Menard D, Jeannoda V (2021). Genetic diversity of *Plasmodium falciparum* populations in three malaria transmission settings in Madagascar. Malar J.

[44] Somé AF, Bazié T, Zongo I, Yerbanga RS, Nikiéma F, Neya C (2018). *Plasmodium falciparum* msp1 and msp2 genetic diversity and allele frequencies in parasites isolated from symptomatic malaria patients in Bobo-Dioulasso, Burkina Faso. Parasit Vectors.

[45] Sondo P, Derra K, Rouamba T, Diallo SN, Taconet P, Kazienga A (2020). Determinants of *Plasmodium falciparum* multiplicity of infection and genetic diversityin Burkina Faso. Parasit Vectors.

[46] Apinjoh TO, Tata RB, Anchang-Kimbi JK, Chi HF, Fon EM, Mugri RN (2015). *Plasmodium falciparum* merozoite surface protein 1 block 2 gene polymorphism infield isolates along the slope of Mount Cameroon: a cross-sectional study. BMC Infect Dis.

[47] Mayengue PI, Ndounga M, Malonga FV, Bitemo M, Ntoumi F (2011). Genetic polymorphism of merozoite surface protein-1 and merozoite surface protein-2 in *Plasmodium falciparum* isolates from Brazzaville, Republic of Congo. Malar J.

[48] Chen J-T, Li J, Zha G-C, Huang G, Huang Z-X, Xie D-D (2018). Genetic diversity and allele frequencies of *Plasmodium falciparum* msp1 and msp2 in parasite isolates from Bioko Island, Equatorial Guinea. Malar J.

[49] Oyedeji SI, Bassi PU, Oyedeji SA, Ojurongbe O, Awobode HO (2020). Genetic diversity and complexity of *Plasmodium falciparum* infections in the microenvironment among siblings of the same household in north-central Nigeria. Malar J.

[50] Oboh MA, Ndiaye T, Diongue K, Ndiaye YD, Sy M, Deme AB (2021). Allelic diversity of MSP1 and MSP2 repeat loci correlate with levels of malaria endemicityin Senegal and Nigerian populations. Malar J.

[51] Robert F, Ntoumi F, Angel G, Candito D, Rogier C, Fandeur T (1996). Extensive genetic diversity of *Plasmodium falciparum* isolates collected from patients with severe malaria in Dakar, Senegal. Trans R Trop Med Hyg.

[52] Ndiaye T, Sy M, Gaye A, Ndiaye D (2019). Genetic polymorphism of merozoite surface protein 1 (msp1) and 2 (msp2) genes and multiplicity of *Plasmodium falciparum* infection across various endemic areas in Senegal. Health Sci.

[53] Ndiaye T, Sy M, Gaye A, Siddle KJ, Park DJ, Bei AK (2020). Molecular epidemiology of *Plasmodium falciparum* by multiplexed amplicon deep sequencing in Senegal. Malar J.

[54] Kaur H, Sehgal R, Goyal K, Makkar N, Yadav R, Bharti PK (2017). Genetic diversity of *Plasmodium falciparum* merozoite surface protein-1 (block 2), glutamate-rich protein and sexual stage antigen Pfs25 from Chandigarh, North India. Trop Med Int Health.

[55] Mohd Abd Razak MR, Sastu UR, Norahmad NA, Abdul-Karim A, Muhammad A, Muniandy PK (2016). Genetic diversity of *Plasmodium*
*falciparum* populations in malaria declining areas of Sabah East Malaysia. PLoS ONE.

[56] Jamil KF, Pratama NR, Marantina SS, Harapan H, Kurniawan MR, Zanaria TM (2021). Allelic diversity of merozoite surface protein genes (msp1 and msp2) and clinical manifestations of *Plasmodium falciparum* malaria cases in Aceh, Indonesia. Malar J.

[57] Zhang C-L, Zhou H-N, Liu Q, Yang Y-M (2019). Genetic polymorphism of merozoite surface proteins 1 and 2 of *Plasmodium falciparum* in the China-Myanmar border region. Malar J.

[58] Congpuong K, Sukaram R, Prompan Y, Dornae A (2014). Genetic diversity of the msp-1, msp-2, and glurpgenes of *Plasmodium falciparum* isolates along the Thai-Myanmar borders. Asian Pac J Trop Biomed.

[59] Khaminsou N, Kritpetcharat O, Daduang J, Charerntanyarak L, Kritpetcharat P (2011). Genetic analysis of the merozoite surface protein-1 block 2 allelic types in *Plasmodium falciparum* clinical isolates from Lao PDR. Malar J.

[60] Long BV, Allen G, Brauny M, Linh LTK, Pallerla SR, Huyen TTT (2020). Molecular surveillance and temporal monitoring of malaria parasites in focal Vietnamese provinces. Malar J.

[61] Pratt-Riccio LR, de Perce-da-Silva D, da Lima-Junior J, Theisen M, Santos F, Daniel-Ribeiro CT (2013). Genetic polymorphisms in the glutamate-rich protein of *Plasmodium falciparum* field isolates from a malaria-endemic area of Brazil. Mem Inst Oswaldo Cruz.

[62] Santamaría AM, Vásquez V, Rigg C, Moreno D, Romero L, Justo C (2020). *Plasmodium falciparum* genetic diversity in Panamá based on glurp, msp-1 and msp-2 genes: implications for malaria elimination in Mesoamerica. Life (Basel).

[63] Federal Ministry of Health of Ethiopia (2012). National malaria guidelines.

