# Supplementary material for: Temporal dynamics of Plasmodium falciparum population in Metehara, east-central Ethiopia
Source: Malar J. 2022 Sep 15;21:267. doi: 10.1186/s12936-022-04277-5 (PMC9479295; doi:10.1186/s12936-022-04277-5)
Supplement: Supplementary file 1 — Additional file 1. Primers used for msp1 genotyping. [file 12936_2022_4277_MOESM1_ESM.docx]

Additional File 1: Primers used for *msp1* genotyping

| Primer | Sequence |
| --- | --- |
| M1-OF | 5'-CTA GAA GCT TTA GAA GAT GCA GTA TTG-3' |
| M1-OR | 5'-CTT AAA TAG TAT TCT AAT TCA AGT GGA-3' |
| M1-KF | 5'-AATGAA GAAGAA ATT ACT ACA AAA GGT-3' |
| M1-KR | 5'-GCT TGC ATC AGC TGG AGG GCTTGC ACC-3' |
| M1-RF | 5'-TAAAGGATG GAGCAAATACTCAAGTTG-3' |
| M1-RR | 5'-CATCTGAAGGATTTGCAGCACCTG GAG-3' |
| M1-MF | 5'-AAA TGAAGGAACAAGTGGAACAGCTGT-3' |
| MI-MR | 5'-ATCTGAAGGATTTGTACGTCTTGAATT-3' |
