# Supplementary material for: Temporal dynamics of Plasmodium falciparum population in Metehara, east-central Ethiopia
Source: Malar J. 2022 Sep 15;21:267. doi: 10.1186/s12936-022-04277-5 (PMC9479295; doi:10.1186/s12936-022-04277-5)
Supplement: Supplementary file 2 — Additional file 2. Primers used for msp2 genotyping. [file 12936_2022_4277_MOESM2_ESM.docx]

Additional File 2: Primers used for *msp2* genotyping

| Primer | Sequence |
| --- | --- |
| M2-OF | 5'-ATGAAGGTAATTAADACATTGTCTATT-3' |
| M2-OR | 5'-CTTTGTTACCATCGGTACATTCTT-3' |
| FC27-B1 | 5'-GCAAATGAAGGTTCTAATACTAATAG-3' |
| FC27-B2 | 5'-GCTTTGGGTCCTTCTTCAGTTGATTC-3' |
| IC/3D7-A1 | 5'-GCAGAAAGTAAG CCTTCTACTGGTGCT-3' |
| IC/3D7-A2 | 5'-GATTTGTTTCGGCATTATTATGA-3' |
