# Supplementary material for: Temporal dynamics of Plasmodium falciparum population in Metehara, east-central Ethiopia
Source: Malar J. 2022 Sep 15;21:267. doi: 10.1186/s12936-022-04277-5 (PMC9479295; doi:10.1186/s12936-022-04277-5)
Supplement: Supplementary file 3 — Additional file 3. Primers used for glurp genotyping. [file 12936_2022_4277_MOESM3_ESM.docx]

Additional File 3: Primers used for *glurp* genotyping

| Primer | Sequence |
| --- | --- |
| G-OF | 5'-TGAATTTGAAGATGTTCACACTGAAC-3' |
| G-OR | 5'-GTG GAA TTG CTTTTTCTTCAACACTAA-' |
| G-NF | 5'-TGAATTTGAAGA TGT TCA CAC TGA AC-3' |
| G-OR | 5'-GTG GAA TTGCTTTTT CTT CAACACTAA-3' |
